# Synchronised monitoring of plant and insect diversity: a case study using automated Malaise traps and DNA-based methods

**DOI:** 10.3897/BDJ.12.e127669

**Published:** 2024-07-30

**Authors:** Leighton J Thomas, Ameli Kirse, Hanna Raus, Kathrin Langen, Björn Nümann, Georg F. Tschan, Birgit Gemeinholzer, J. Wolfgang Wägele, Sarah J Bourlat

**Affiliations:** 1 Leibniz Institute for the Analysis of Biodiversity Change, Museum Koenig, Bonn, Germany Leibniz Institute for the Analysis of Biodiversity Change, Museum Koenig Bonn Germany; 2 University of Kassel, Kassel, Germany University of Kassel Kassel Germany

**Keywords:** metabarcoding, plant traces, plant-insect interaction, automated Sampling, Malaise trap, biomonitoring, phenology

## Abstract

The occurrence and distribution of insects and their possible associations with plant species are largely unknown in Germany and baseline data to monitor future trends are urgently needed. Using newly-designed automated Malaise trap multi-samplers, the occurrence of insect species and their potential associations with plants was monitored synchronously at two contrasting field sites in Germany: an urban botanical garden and a forest research station. Taxa were identified by metabarcoding of the insects and the plant traces present in the preservative ethanol of the Malaise trap samples. For comparison, a botanical survey was conducted in the vicinity of the traps. Across both sites, we identified a total of 1290 exact sequence variants (ESVs) assigned to Insecta, of which 205 are known to be pollinators. In the botanical garden, we detected the occurrence of 128 plant taxa, of which 41 also had one of their known insect pollinator species detected. Insect species richness was highest in May, mainly attributed to an increase in Diptera. These results present a case study of the applicability of automated sampling and DNA-based methods to monitor the timings of flowering and corresponding activity of plant-visiting insects.

## Introduction

A comprehensive understanding of insect diversity and distributions requires consistent and standardised monitoring. However, monitoring of insects is currently hindered by the complexity and time-consuming nature of species identification, which is further impeded by a decline in trained taxonomists (Engel et al. 2021). Additional challenges include: (1) insects are a highly diverse group with large differences in species diversity over small spatial scales; (2) the necessity of substantial, standardised and coordinated efforts to enable temporal comparisons across different regions (see, for example, Hausmann et al. (2020)); (3) fieldwork and species identification present a substantial workload and are the most time-consuming aspect of insect monitoring; (4) the absence of quality-controlled open-access systems for monitoring data; (5) the lack of baseline reference data; (6) The lack of complete and curated barcode reference databases serve as a hindrance to effective insect monitoring. To address some of these problems, it is vital to develop a biodiversity monitoring system that is deployed over large spatial scales, covering diverse habitats and which is synchronised in time. To be feasible, these workflows must be standardised and largely automated for continuous monitoring of biodiversity, analogous to the methods established for climate research.

Despite their well-established importance, concerns still persist regarding the decline of pollinator and plant populations (Goulson et al. 2015, Genung et al. 2022). Over the past two centuries, Europe has witnessed the extinction of numerous bee, wasp and butterfly species (Ollerton et al. 2014), resulting in a parallel decline of their associated plants (Biesmeijer et al. 2006). The decline in pollinator species is particularly pronounced for species that rely on late summer flowering plants (Balfour et al. 2018). Despite the importance of interactions between insects and plants, our understanding of the specific plant preferences of many insect species remains limited. It is now well known that climate change is causing phenological shifts, with plants demonstrating more pronounced shifts than insects (Vitasse et al. 2022). Many plants are now flowering earlier, raising a concern that such phenological shifts will lead to a mismatch in the insect pollinator activity (Memmott et al. 2007, Duchenne et al. 2019). Since the beginning of scientific recording, pollinators in Germany have been active before the onset of flowering, yet the faster shift of plants has resulted in an increase in synchrony. However, if the phenological shifts continue in line with climate warming, they are likely to result in greater asynchrony (Freimuth et al. 2022).

Here, we use automated Malaise trap samplers to monitor insect and plant diversity over time, as described in Wägele et al. (2022) and Wägele and Tschan (2024b). The ‘Automated Multisensor stations for Monitoring of bioDiversity’, also known as AMMODs, are newly-designed biodiversity monitoring stations, which include devices for airborne pollen sampling, an automated Malaise trap, volatile organic compound detection, a camera trap, bioacoustic recording and an integrated weather station. Here, we focus on the automated Malaise trap (hereafter referred to as ‘insect sampler’) (Wägele and Tschan 2024b). The insect sampler consists of an automated bottle changing mechanism attached to a standardised Malaise trap which can collect up to 12 individual insect bulk samples in programmable intervals, and it is equipped with a communication system which updates the user on the system status (Kirse et al. 2024). The traps were synchronised between sites, allowing temporal comparison of insect communities. Metabarcoding allowed to not only identify the insects caught in the traps, but also the pollen and other plant traces carried by the insects, which were extracted from the preservative ethanol in the samples.

In this study, we focus on the detection of insects and their associated plants, greatly increasing the knowledge of local species occurrences. Since the data have a temporal component, information on insect flight times can also be obtained. The co-occurrences of plant traces and insects can be traced across a temporal scale, making it possible to establish a baseline dataset of flower-visiting insect flight times for future comparisons. Using these newly-developed tools, we sought to answer the following questions: which plant species can be detected from pollen and other plant traces accumulated in the insect samples? Do the detected plants match existing species lists from the vicinity of the traps? Do temporal patterns of insect occurrence correspond to the detection of individual plant species as recorded in the database of pollinator interactions (DoPI, Balfour et al. 2022)? The results presented here demonstrate a proof of concept for combining plant and insect monitoring technologies. In this article, we examine the potential benefits and shortcomings of the system.

## Methods

### Study sites and sampling

The insect samplers are Malaise traps complemented with an automated multisampler, containing 12 sample bottles and a control unit. The sample vials can be changed automatically following predetermined time settings (see Kirse et al. (2024) for further details). The insect samplers were used at two field sites: (a) the Melbgarten, a subsidiary site of the University of Bonn Botanic Gardens in Bonn, Germany (50.712845°N, 7.09026°E); (b) a forest plot of the nationwide forest monitoring programme at the Thünen Institute in Britz, Germany (52.87826°N, 13.8333°E). The Melbgarten partially borders both a nature reserve (‘Naturschutzgebiet Melbtal’) and a residential area; its terrain slopes slightly to the west. The Britz site is a four-hectare ecological research station of the Thünen Institute in Britz near Eberswalde, which is mainly surrounded by pine forests, about 400 m from the nearest village. Both sites are completely enclosed by a fence (Wägele and Tschan 2024a). Insects and their associated plant traces (presumably consumed plant material or externally attached pollen traces) were sampled using the insect sampler. The times and dates for automated rotation of the collection bottles were synchronised at both locations, which allowed temporal comparison of the samples. Sample collection was conducted from late March 2022 to mid-September 2022, with a two-week interval for changing of the bottles. This resulted in 12 samples for the Britz site and five samples for the Melbgarten site, due to a malfunction of the system at the latter (Table 1).

### Botanical survey of sites

The Melbgarten contains both planted and naturally occurring species, which are well documented. The planted material originates predominantly from Georgia and China, but large parts of the garden are not actively cultivated. A comprehensive list of all of the planted species in the Melbgarten was provided by the curators (Supplementary Table 2). Naturally occurring species within a five-metre radius around the traps were identified using standard botanical identification literature, supported by the identification apps ‘ObsIdentify’ (Observation International 2024; see also Schermer and Hogeweg (2018)) and ‘Flora Incognita’ (Flora Incognita 2024). Identifications were checked for plausibility by comparing existing species lists for the garden and with occurrence data in the Global Biodiversity Information Facility (GBIF) data. It was not possible to obtain a pre-existing species list of the plants occurring at the Britz site; therefore, only data from plant occurrences documented in GBIF for the wider local administrative region were used. The species lists obtained via metabarcoding were later compared to these lists.

### Plant metabarcoding

#### DNA extraction, PCR, Illumina amplicon library preparation and sequencing

Following removal of insects by sieving, the preservative ethanol from the Malaise trap samples was vacuum filtered using a cellulose nitrate membrane (GVS Filter Technology, Sanford, USA; diameter 47 mm and 0.22 μl pore size). After filtration, the cellulose nitrate filter was cut in half and each half was placed in a separate 2 ml SafeSeal micro tube (Sarstedt AG & Co. KG, Nümbrecht, Germany). One half of the filter paper was used for DNA isolation, the other half was kept as a voucher.

DNA extraction from the plant parts consumed or pollen externally attached to insects found in the preservative ethanol of the Malaise trap was performed with the NucleoMag Plant Kit (Macherey-Nagel, Düren, Germany). Two DNA extraction negative controls were processed for each batch of up to 24 samples. The detailed protocol for DNA extraction from plant material, as well as clean lab methods used for quality assurance, are described in Raus et al. (2024). DNA extracts from plant traces extracted from the preservative ethanol were submitted to the LIB ZFMK biobank under ZFMK-DNA-Bank Box number ZFMK-DNA-Bank_1905_SA00936025 for long-term storage.

The ITS2 region was used to generate amplicon libraries using a dual PCR protocol in which the barcode region was amplified in the first round of PCR and the flow cell binding adapter, a sequencing primer binding site and an index were attached in the second round of PCR (Raus et al. 2024). PCR was performed with the following primers (ITS-3p62plF1: ACBTRGTGTGAATTGCAGRATC and ITS-4unR1: TCCTCCGCTTATTKATATGC) proposed by Kolter and Gemeinholzer (2021). Two blank DNA extractions and two PCR negative controls were added to every 96 well PCR plate. Furthermore, a mock community consisting of five replicates of five different species that do not occur in the study area (*Clerodendrumthomsoniae*, *Coffeaarabica*, *Euphorbiamollis*, *Sophoratetraptera*, *Spathiphylum* sp.) was added as a positive control and quality filter. PCR was performed in triplicates as described in Raus et al. (2024). Initial denaturation was performed at 95°C for 3 minutes, followed by 30 cycles of denaturation at 95°C for 30 seconds, annealing at 50°C for 30 seconds and extension at 72°C for 45 seconds. The final extension was performed at 72°C for 8 minutes. After PCR, all three PCR replicates of a sample were combined and purified with Exonuclease 1 (Thermo Fisher Scientific, Waltham, USA) following the manufacturer's instructions. Illumina amplicon library preparation was performed with 12 additional cycles at LGC Genomics GmbH (Berlin) and sequencing was carried out on a MiSeq platform (2 x 300 bp, Illumina, San Diego, USA). For the additional PCR cycling MyTaqTM Red Mix polymerase (Meridian Bioscience, Cincinnati, USA) was used and consisted of three cycles with a low annealing temperature (15 sec 96°C, 30 sec 50°C, 90 sec 70°C), followed by nine cycles with increased annealing temperature (15 sec 96°C, 30 sec 58°C, 90 sec 70°C). Raw sequence reads are stored at the GenBank SRA database under accession number PRJNA1068928.

#### Bioinformatics and taxonomic assignment

Demultiplexing and primer removal was performed using Cutadapt v.1.9.1 (Martin 2011). Subsequently, sequences were quality filtered with FastQC (Andrews 2014) and then processed and merged via the R package DADA2 version 1.16 (Callahan et al. 2016). The resulting exact sequence variants (ESV) were taxonomically assigned using the PLANiTS2 database (Banchi et al. 2020), using the DADA2 assignTaxonomy function (Callahan et al. 2016) (see Suppl. material 2 for the ESV table). Fungal contamination was confirmed and removed using a local BLASTn search and a custom fungal ITS-BLAST database (Camacho et al. 2009). The reads found in the negative controls were subtracted from the ESVs of all other samples to reduce the effect of contamination. ESVs identified as fungi, algae and lichens were removed from the plant dataset. ESVs with ambiguous species identifiers received only genus-level identifiers. ESVs from the DNA extraction and PCR blanks were used to calculate a relative abundance and ESVs below this threshold were removed. Subsequently, the ESV table was converted to a presence/absence matrix.

### Insect metabarcoding

#### Size sorting and DNA extraction

Preservative ethanol was removed from the Malaise trap samples and the ethanol stored for the subsequent extraction of plant material. Insects were sieved using a 4 x 4 mm mesh (wire diameter 0.5 mm, untreated stainless steel), resulting in two size fractions: S (small, ≤ 4 mm) and L (large, > 4 mm). Individuals of both size fractions were transferred to either disposable grinding chambers (IKA, 40 or 100 ml) or 30 ml Nalgene tubes with metal beads (5 mm in diameter) and dried in an incubator at 50°C for up to 5 days until complete ethanol evaporation. Dried insect tissue was homogenised for 3 minutes either with a batch mill (Tube Mill 100 Control, IKA) at 25,000 rpm or a mixer mill (MM400, Retsch) at 30 Hz for 5 minutes. Approximately 20 mg of finely homogenised tissue were transferred to a 1.5 ml Eppendorf tube and 190 µl of ATL buffer (Qiagen, Hilden, Germany) and 10 µl of Proteinase K (Qiagen, Hilden, Germany) were added. The samples were incubated overnight at 56°C using a shaking incubator to allow for tissue lysis (110 rpm, INCU Line ILS 6, VWR, Radnor, PA, USA). Twelve negative controls containing 200 μl of ATL buffer only were added for each batch of 84 samples during processing in 96 well plate format. DNA was extracted from the Melbgarten samples for each size fraction (S and L) separately with the DNeasy 96 Blood and Tissue Kit (Qiagen, Hilden, Germany) following the manufacturer's instructions. For the Britz samples, 135 µl of the S fraction lysate and 15 µl of the L fraction lysate of each sample were merged according to Elbrecht et al. (2021) before proceeding with DNA extraction with the DNeasy 96 Blood and Tissue Kit following the manufacturer's instructions. Extraction success and DNA quality were checked on a 1% agarose gel. Ground tissue samples and DNA extracts, as well as extensive sample metadata, were submitted to the LIB ZFMK biobank and are stored under accession numbers ZFMK-TIS-78623 to ZFMK-TIS-78646.

#### PCR, Illumina amplicon library preparation and sequencing

The dual PCR protocol of Bourlat et al. (2016) was used for amplicon library preparation. The first PCR contained 12.5 µl master mix (PCR Multiplex Plus Kit, Qiagen, Hilden, Germany), 1 μl template DNA, 0.2 µM fwhF2 forward primer (GGDACWGGWTGAACWGTWTAYCCHCC) (Vamos et al. 2017), 0.2 µM FolDegenRev reverse primer (TANACYTCNGGRTGNCCRAARAAYCA) (Yu et al. 2012) and ddH_2_0 to make up a 25.0 μl final reaction volume. The primer pair targets a 313 bp long stretch of the COI DNA barcode region and ensures sufficient overlap of fragments during paired-end merging after 2 x 250 bp sequencing. The PCR programme was run as follows on a 2720 187 Thermal Cycler (Applied Biosystems): initial denaturation at 95°C for 5 min; 25 cycles of: 30 s at 95°C, 30 s at 50°C and 50 s at 72°C; final extension of 5 min at 72°C. In the second PCR, combinatorial dual indexing using a set of 16 forward and 24 reverse primers with unique identifiers was used to guarantee the assignment of sequences to the sample of origin. The reaction included 1 μl PCR 1 product template, 0.2 µM of each tagging primer (Nextera, Illumina, San Diego, USA), 12.5 μl master mix (PCR Multiplex Plus Kit, Qiagen, Hilden, Germany) and 9.5 μl ddH_2_O. PCR 2 was run with the same programme as PCR 1, but with 15 instead of 25 cycles.

PCR success was checked on a 1% agarose gel before PCR products were normalised using a SequalPrep normalisation plate (Thermo Fisher Scientific, MA, USA) following the manufacturer’s instructions, resulting in a final DNA yield of 25 ng per sample (20 μl volume). For each sample, 10 μl aliquots were pooled and two rounds of left-sided size selection were carried out on the sample pool with magnetic beads to remove primer dimers (ratio 1:0.7, SPRIselect Beckman Coulter). Library concentration was measured with a Quantus fluorometer (Promega, Madison, USA) and on a Fragment Analyzer (Agilent Technologies, Santa Clara, CA, USA). The pool was sent for sequencing on two Hiseq 2500 runs (2 x 250 bp) (Macrogen Europe B.V., Netherlands). Raw data were uploaded to the GenBank SRA archive under bioproject accession number PRJNA1068928.

#### Bioinformatics and taxonomic assignment

Following the APSCALE pipeline (Buchner et al. 2022), demultiplexed reads were pair-aligned with VSEARCH (Rognes et al. 2016) and primers were removed using CUTADAPT (Martin 2011). Reads were then filtered, based on per-base quality using VSEARCH, using the following settings: maximum expected errors = 1, minimum length = 310, maximum length = 320. Sequence denoising is based on the alpha value (using default value = 2), which corresponds to the number of allowed sequence differences (Edgar 2016). Before taxonomic assignment, the resulting ESV tables were filtered for potentially biased sequences using the LULU algorithm (Frøslev et al. 2017), which reduces erroneous ESVs while retaining rare, but real ESVs by merging “daughter” ESVs with consistently co-occurring, but more abundant ‘parent’ ESVs. We annotated representative sequences with taxonomic names using the Python package BOLDigger (Buchner and Leese 2020) which references public and private data in the Barcode of Life Database (BOLD) (Ratnasingham and Hebert 2007). Abundance-based filtering was carried out to remove all ESVs which were less than 0.001% of the read count per sample with subsequent removal of remaining singletons. The maximum read count of each ESV found in the negative controls was subtracted for all the samples to remove the effect of unwanted contamination. Taxa that had less than a 99% similarity to the database assignment were not included in further analysis; this meant that all ESVs were of high confidence and all were all assigned to species level taxonomy (see Suppl. material 1). In order to review the presence of potential pollinator interactions, the insect species detected in our study were then compared to the database of pollinator interactions (DoPI) (Balfour et al. 2022). The species occurrences in each sampling interval were plotted using the R package *upsetR* in combination with the package *ComplexUspetR* which is an extension of the Venn diagram for multiple sets (Fig. 2).

## Results and species lists

### Multisampler sampling

Due to a malfunction of the insect multisampler at the Melbgarten sampling site, only eight pollen and five insect samples were reliably collected. From sampling interval 9 onwards (see Table 1 for sampling dates), there was a malfunction in the stop mechanism of the rotating sampler. For this reason, subsequent samples after interval 8 were not included in further analyses. During the collection period, the outside temperature was so high that the preservation ethanol (500ml) evaporated and the insects ultimately remained dry in some of the samples, resulting in less reliable insect metabarcoding anayses. For this reason, insect samples after interval 5 in the Melbgarten (Bonn) were excluded from the analysis. No reduction in species richness was observed in the plant traces in the insect multisampler samples, so the resulting species lists were retained for analysis of the data. This left a total of eight bottles which were metabarcoded for plant traces and five which were metabarcoded for insects at the Melbgarten. At the Britz site, the multisampler functioned as expected, yielding 12 bottles metabarcoded for both plant traces and insects (Table 1).

### Plant species identification

A total of 163 ESVs detected in the Britz site were assigned to 41 families, of which 45 could only be determined to genus level and one could only be assigned to family level, as polyploidy and hybridisation in *Hieracium* and *Pilosella* hindered unambiguous genus assignment. A total of 128 ESVs representing 37 different plant families were recorded at Melbgarten, of which 31 could only be determined to genus level and one could only be assigned to family level (Suppl. material 2). The most commonly detected families were: Asteraceae (Britz: 19 ESVs, Melbgarten: 9 ESVs), Poaceae (Britz: 19 ESVs, Melbgarten: 8 ESVs), Rosaceae (Britz: 17 ESVs, Melbgarten: 6 ESVs) and Brassicaceae (Britz: 13 ESVs, Melbgarten: 6ESVs), Ranunculaceae (Britz: 6 ESVs, Melbgarten: 9 ESVs), Hydrangeaceae (Britz: 2 ESVs, Melbgarten: 9 ESVs), Apiaceae (Britz: 4 ESVs, Melbgarten: 8 ESVs), Fabaceae (Britz: 7 ESVs, Melbgarten: 9 ESVs) and Poaceae (Britz: 19 ESVs, Melbgarten: 8 ESVs). Only ESVs that were assigned to at least genus level were retained for subsequent analysis.

### Insect species identification

Across the two sampling sites, a total of 1290 ESVs assigned to Insecta were detected (Suppl. material 1). As we used a strict similarity setting (> 99%), all ESVs were given a species level taxonomic name. Diptera and Hymenoptera made up the majority of species detected in the traps (Diptera 715 ESVs, 55%), (Hymenoptera 179 ESVs, 13%), followed by Coleoptera (161 ESVs, 12%), Hemiptera (106 ESVs, 8%) and Lepidoptera (74 ESVs, 5%).

### Pollinator species and their known interactions

Across both sites, a total of 205 insect species recognised as pollinators were identified (Suppl. material 1) of which the hoverfly *Rhingiarostrata* and the solitary bee *Megachilemaritima* are classified as endangered on the German Red List. At Melbgarten, 124 pollinator species (Suppl. material 1) and 128 plant taxa (Suppl. material 2) were identified, of which 41 species are associated with an insect pollinator (according to DoPI, Balfour et al. (2022)) which, in most cases, were also detected through metabarcoding (Suppl. material 3, Fig. 4 and Table 2). Thirty of the plants identified through metabarcoding were confirmed to exist in the Melbgarten as they were either known to be planted or were identified by two of the authors during an on-site survey. Another 42 plant taxa, not known to exist in the Melbgarten, have known occurrence data on GBIF and, finally, there were 51 species that did not have previous occurrence data. At Britz, 122 insect pollinator species (Suppl. material 1) and 163 different plant taxa (Suppl. material 2) were detected, of which 55 had known insect pollinators (according to DoPI, Balfour et al. (2022)) that were detected through metabarcoding (Suppl. material 4, Table 2, Fig. 4). Most of the plant species detected were expected given known occurrences in the Brandenburg administrative region in GBIF. We would like to clarify here that it is not possible to reconstruct plant-insect interactions at the level of single insects with this method and that all interactions mentioned here are known interactions inferred from the database of pollinator interactions (DoPI, Balfour et al. (2022)).

### Temporal comparisons of pollinator species richness between sites

**Figure 1.**
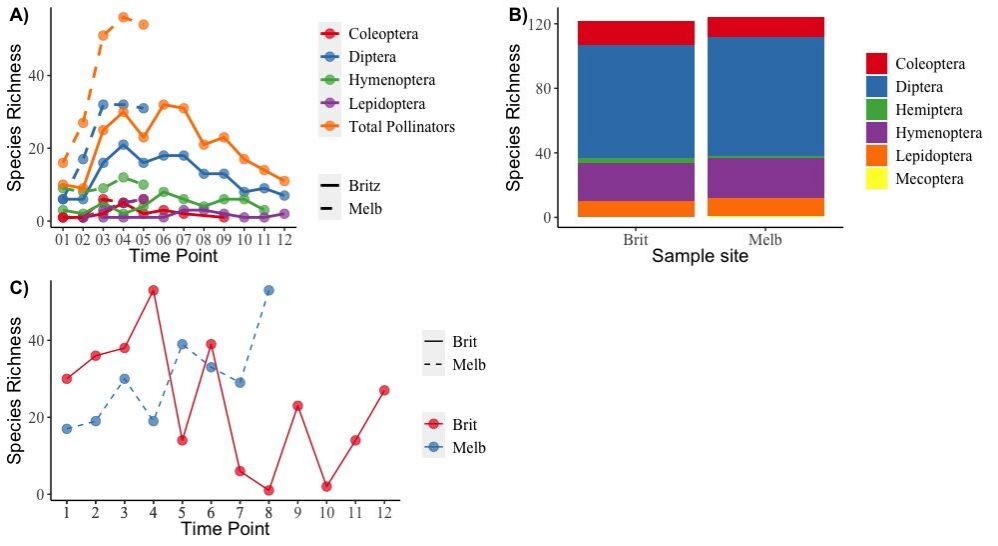
**A)** Total pollinator species richness (orange) across time for both Britz (sampling points 1-12) and the Melbgarten (sampling points 1-5). Species richness across time per order is presented for Coleoptera (red), Diptera (blue), Hymenoptera (green) and Lepidotera (purple); **B)** Community composition bar plot showing the number of pollinator species per order at both sampling sites; **C)** Taxon richness across time for plants detected in the insect multisampler.

**Figure 2.**
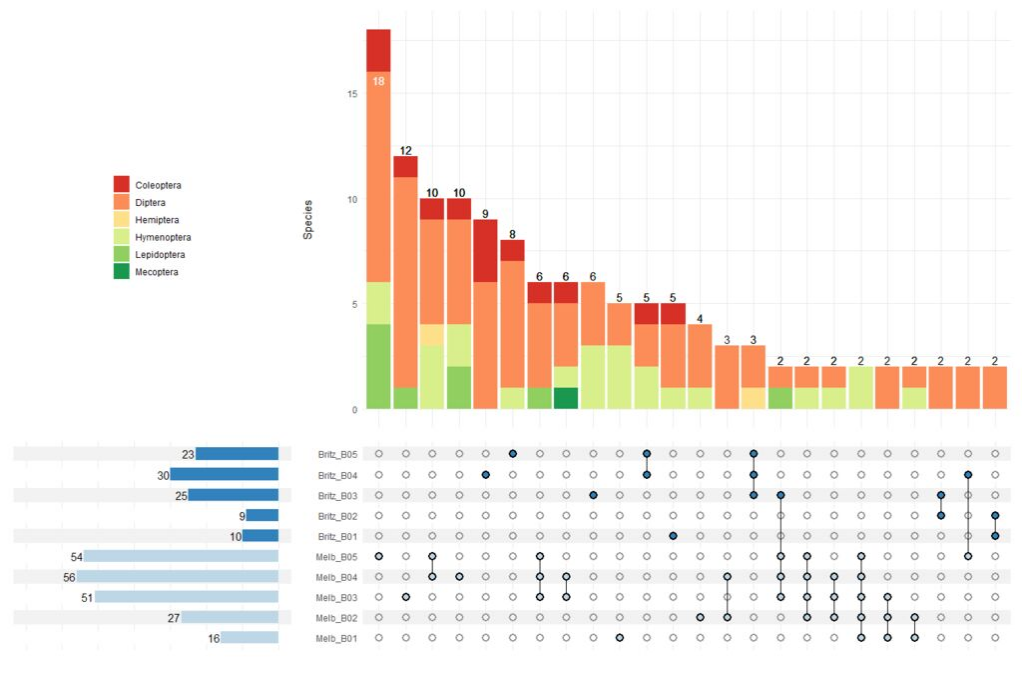
UpsetR plot showing the species overlap between sampling points for the insect pollinators. Horizontal bars on the left indicate the total number of detected insect pollinator species per sampling point at Britz and the Melbgarten (Melb). Vertical bars indicate the number of shared and unique species within and between sampling points, as well as their taxonomic composition.

**Figure 3.**
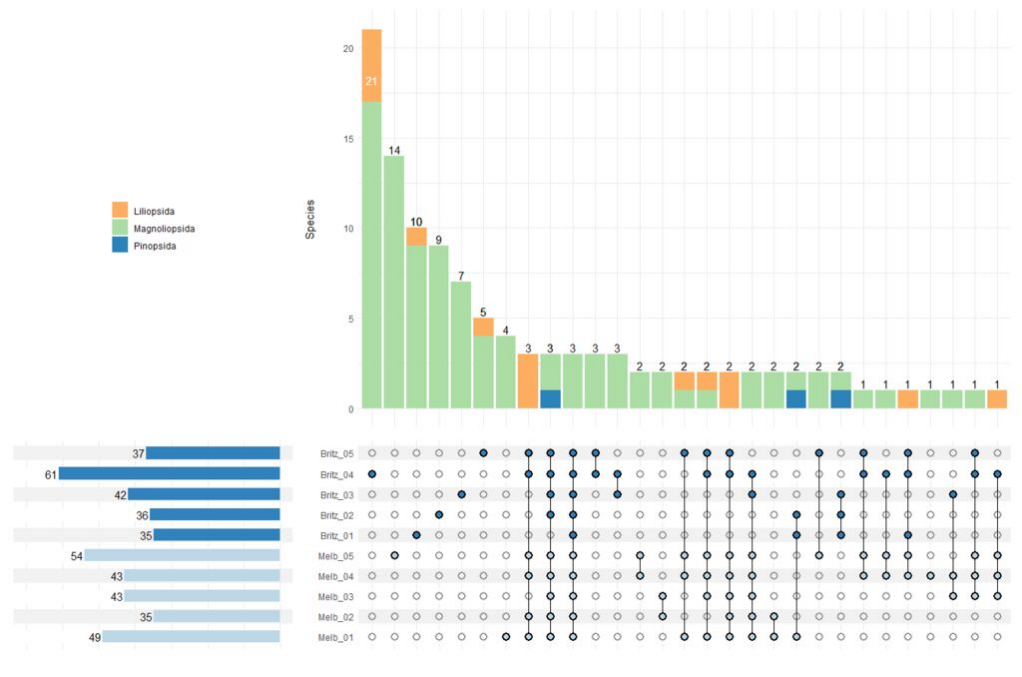
UpsetR plot showing the overlap between sampling points for the plants found in the Malaise trap samples. Horizontal bars on the left indicate the total number of detected plant taxa per sampling point at Britz and the Melbgarten (Melb). Vertical bars indicate the number of shared and unique taxa within and between sampling points, as well as their taxonomic composition.

Peak pollinator species richness occurred at sampling round 4 (12 May 2022 – 26 May 2022) in both locations with species richness overall higher in the Melbgarten (Fig. 1A). Diptera formed the largest group of pollinators detected at both sites (Fig. 1B) and the temporal increase in species richness in sampling rounds 1 to 4 was most pronounced for this group in comparison to other orders (Fig. 1A). Similarly, peak plant species richness detected in the insect multisampler occurred at sampling round 4 (12 May 2022 – 26 May 2022) in the Britz site and at sampling round 8 (07 July 2022 – 22 July 2022) in the Melbgarten site (Fig. 1C). The pollinator species found at each sampling interval demonstrate very high levels of uniqueness between samples in both Britz and the Melbgarten, with the majority of species detected in one sampling round, suggesting phenological effects (Melb_B05 and Melb_B03, see Fig. 2). In the Melbgarten there are only two pollinator species occurring in all sampling rounds (Fig. 2). A similar pattern is revealed with the plants with high numbers of species detected in a single sampling interval (Fig. 3). At the sampling site in Britz, 21 species belonging to the classes Magnoliopsida and Liliopsida were only detected in sampling interval Britz_04 (Fig. 3), while 14 unique species of Magnoliopsida were detected in the insect multisampler in sampling interval Melb_05 in the Melbgarten (Fig. 3).

## Discussion

**Figure 4.**
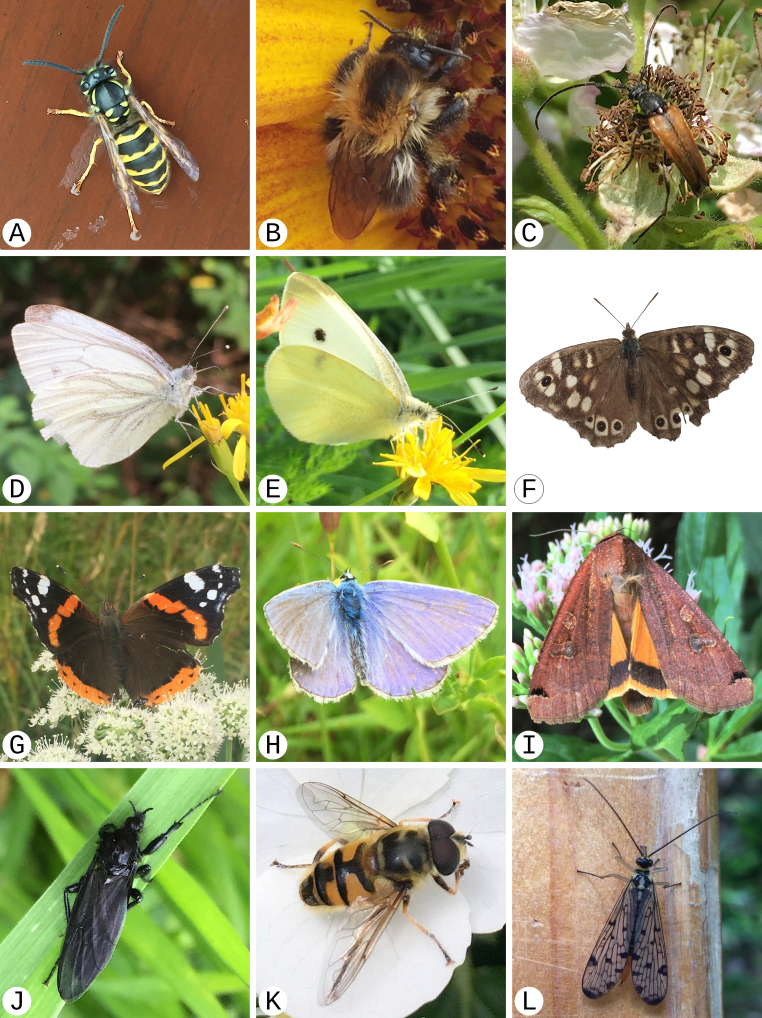
Examples of the insect species found in the Malaise trap sampling of this study that are also pollinators according to the Database of Pollinator Interactions (DoPI) (Balfour et al. 2022). Image collection G. F. Tschan. The photographs were taken in western and southern Germany between April 2022 and September 2023. All identifications were confirmed using the application for automated image recognition ‘ObsIdentify’ (Observation International 2024). **A** *Vespulavulgaris*; **B** *Bombuspascuorum*; **C** *Stenurellamelanura*; **D** *Pierisnapi*; **E** *Pierisrapae*; **F** *Parargeaegeria*; **G** *Vanessaatalanta*; **H** *Polyommatusicarus*; **I** *Noctuapronuba*; **J** *Bibiomarci*; **K** *Myathropaflorea*; **L** *Panorpagermanica*. For further information, see Table 2.

In temperate regions, 78% of all flowering plants are animal-pollinated, of which most are insects (Ollerton et al. 2011). This figure alone illustrates the importance of insects, although it covers only one aspect of the many plant-insect interactions. Today, there are still many gaps in our knowledge of plant-insect interactions. In part, this can be attributed to a focus on charismatic species such as butterflies, bees and hoverflies compared to other important, but less charismatic pollinating insects, such as the Diptera (Davis et al. 2023). Nevertheless, even many Diptera can be observed relatively easily, such as *Myathropaflorea* (Fig. 4K), which prefers large flowers, for example, of the plant families Apiaceae or Asteraceae.

Recording the interactions between plants and insects in the field can be tedious. In addition, the quality of the data collected depends on the training and working accuracy of each individual involved. The use of the automated insect multisamplers combined with metabarcoding techniques is a contribution to reducing sampling effort and standardising the acquisition of temporal monitoring data, in a user-independent way. In the AMMOD project, plant species were detected using: (i) airborne pollen traps (Raus et al. 2024) and (ii) through the detection of plant volatile organic compounds (pVOCs) in ambient air (Losch et al. 2024); (iii) the automated Malaise traps used in this study provided additional records of plants detected in direct association with the insects sampled. We wish to point out here that the plant traces detected in the insect multisampler could be present due to the following reasons: (i) they were carried in, attached to flower-visiting insects; (ii) they are ingested food material from insect herbivores or (iii) they were carried in by aerial transport or ambient contamination.

The unveiling of plant-insect interactions using DNA-based methods is an emerging field (Banerjee et al. 2022). In order to understand plant-insect interactions in the absence of direct observation (which would be, for example, manual sorting by researchers or the use of camera traps), each insect would need to be barcoded separately, along with its pollen load and gut contents or eDNA traces would have to be extracted from flowers (Newton et al. 2023). This would generally only be feasible for small sample sizes. Our own study is based on only two sampling sites, where the automated insect multisampler allowed for large, continuous samples sizes, for which the human effort needed was minimal. Nevertheless, we wish to stress that it is not possible to reconstruct interactions at the level of single insects with the methods presented here. In addition, the plant traces detected in the Malaise trap are not necessarily derived from pollination activity, but can be due to feeding activities (e.g. on plant sap or nectar), airborne pollen contamination from ambient air or simply flower visitation.

Some of the co-occurrences can be revealed indirectly using statistical methods, for example, with the R package *cooccur* (Griffith et al. 2016), which calculates the probability of both observed and anticipated frequencies of co-occurrence between every pair of two species. This analysis produces probabilities of the likelihood of observing a particular level of co-occurrence by chance. These occurrences are grouped into the following categories: (i) positive (co-occurring at higher than expected by chance); (ii) negative (co-occurring at a lower probability than expected by chance) and (iii) random (the calculated probabilities are random or data deficient). Given the preliminary nature of this pilot study and the small sample size, this approach could not be used reliably for our dataset.

Some interactions between plants and insects are already well known (DoPI, Balfour et al. (2022)) and it is quite possible to deduce these relationships from the species composition in our Malaise trap. Of the depicted species, the common carder bee, *Bombuspascuorum* (Fig. 4B), can choose from a relatively wide range of food plants in comparison to other species of the genus *Bombus* in Central Europe, which is due to a greater variability in terms of body size and a longer proboscis; it is, therefore, also less dependent on specific plant communities than other species (Kratochwil and Schwabe 2001). In contrast, the common blue, *Polyommatusicarus* (Fig. 4H), has a relatively small food-plant tolerance and is dependent on certain fallow stages of semi-arid grasslands. The complexity of interactions and variety of habitats needed can be illustrated by the speckled wood, *Parargeaegeria* (Fig. 4F), whose egg and caterpillar develop on a variety of grasses, but whose imago lives along forest edges or paths, in clearings and on bushes. All of these habitats are transitional areas between different biological communities and represent micromosaic structures of the local habitat, which are decisive for the species’ existence. Finally, the red admiral, *Vanessaatalanta* (Fig. 4G), also inhabits ecotones, particularly south-facing transitional habitats between open and more closed vegetation; yet interestingly, it is one of a number of native species that also visit introduced plants, so-called neophytes (Kratochwil and Schwabe 2001).

These examples emphasise the complexity of the interaction between plants and insects, some of which are known, but many of which have yet to be investigated. Until recently, it was not even clear how many of the more than 350,000 angiosperm species interacted with pollinators, with figures ranging from less than 70 and close to 100 percent; for temperate-zone communities, the proportion is – on average – a bit more than three quarters (Ollerton et al. 2011). Yet, for particular examples, it is still not always clear whether a plant can be classified as insect- or wind-pollinated; for example, wind-pollinated plants have been shown to be important dietary and nectar resources for insect pollinators, which has been demonstrated for bees and syrphid flies (Saunders 2017).

In our samples, there was a low degree of overlap between insects detected in each time-frame, possibly due to short species-specific flying time-frames of plant-visting insects. The low degree of overlap is a promising indication that the new methodologies can be used to determine flight and flowering times with a higher degree of precision. However, this phenomenon is also a characteristic of Malaise traps: it has been demonstrated that – even when placed in close vicinity – there is still a low degree of overlap between the species caught in adjacent traps (Steinke et al. 2021). The flying activity of pollinating insects has been shown to coincide with the flowering phenology of their associated hosts; for example, British pollinating species (such as aculeate wasps, bees and butterflies) have peak observations in July and August (Balfour et al. 2018). The authors demonstrated that there was a peak in species richness of hoverflies around May, which is late boreal spring and coincides with the flowering of most tree species in this region. A similar pattern for species richness was detected for Dipteran pollinators, which peaked in May, then flattened later in the year. As a result, the relationship between insect activity and plant flowering phenology should be documented over a wide spatial and temporal scale, because ongoing changes to the climate have likely already shifted pollinator phenology over the years (Bartomeus et al. 2013).

The methodologies described here provide an example of how trends could be monitored in the future. For example, the first and last seasonal detection of insect and plant taxa could be used to estimate flight onset and duration for floral visitors, as well as peak flower visiting periods. Additionally, a greater understanding of the biology of insects needs to be considered and, here, the data can be used to elucidate different phenological patterns. For example, *Andrenacineraria* (Hymenoptera) has multiple generations per year, but in our study, the species was detected in only one time interval at both sites; it might perhaps be detected for each of its generation in a given year if monitoring occurred over longer temporal scales. The example also shows that the methods must be standardised and the instruments calibrated. Nevertheless, long term monitoring could, indeed, be used to investigate phenological changes triggered by climate change.

Consistent and standardised methods for monitoring populations are needed for the assessment of extinction risk faced by invertebrate species (Akçakaya et al. 2021). Currently, there is a clearly noticeable lack of long time-series observations for insect populations, which is impeding our knowledge of current insect occurrence and distributions (Didham et al. 2020). A greater understanding of pollinator interactions can allow for better conservation management practices. A better grasp of specific, particularly endangered, plant-insect associations is also needed in order to choose the right conservation rehabilitation strategies. If applied on a larger scale, encompassing a broad range of spatial and temporal dimensions, the methodologies presented in this study can be employed to monitor the fluctuations in the flight times of flower-visting insects, providing documentation of shifts influenced by climate change.

## Supplementary Material

7D5EA2B4-2ACC-5472-98E8-1B8160EE8C25 10.3897/BDJ.12.e127669.suppl1 Supplementary material 1 Insect ESVs detected in the insect malaise trap multisampler Data type ESV table Brief description Presence/absence of insect ESVs detected in the insect malaise trap multisampler in samples from the Melbgarten and Britz sites. Percentage similarity to BOLD records, Red List category and invasive or pollinator status of the species are also shown. File: oo_1011599.xlsx https://binary.pensoft.net/file/1011599 Thomas, L.

DA97D496-34F6-56A5-8A15-2437B2858744 10.3897/BDJ.12.e127669.suppl2 Supplementary material 2 Plant ESVs detected in the insect Malaise trap multisampler Data type ESV table Brief description Presence/absence of plant ESVs detected in the insect Malaise trap multisampler in samples from the Melbgarten and Britz sites. File: oo_1011622.xlsx https://binary.pensoft.net/file/1011622 Raus, H.

DFBE9E05-8EB2-5757-A2E9-786D6C8FA4BD 10.3897/BDJ.12.e127669.suppl3 Supplementary material 3 Plant-pollinator interactions found in the Melbgarten Data type Occurrence data Brief description This table shows the plant-pollinator interactions found in the Melbgarten. In order to review the presence of known pollinator interactions, the insect species detected in our study were compared to the database of pollinator interactions (DoPI) (https://www.sussex.ac.uk/lifesci/ebe/dopi/) (Balfour et al. 2022). File: oo_1025670.xlsx https://binary.pensoft.net/file/1025670 Thomas, L.

CC00F4EE-8A95-5313-96E6-803B5614156A 10.3897/BDJ.12.e127669.suppl4 Supplementary material 4 Plant-pollinator interactions found in Britz Data type Occurrence data Brief description This table shows the plant-pollinator interactions found at the Britz site. In order to review the presence of known pollinator interactions, the insect species detected in our study were compared to the database of pollinator interactions (DoPI) (https://www.sussex.ac.uk/lifesci/ebe/dopi/) (Balfour et al. 2022). File: oo_1025678.xlsx https://binary.pensoft.net/file/1025678 Thomas, L.

## Figures and Tables

**Table 1.** The samples collected using the insect multisampler. Due to a malfunction of the Malaise trap at the Melbgarten collection site, only samples B01 to B08 were collected. Of these samples, B01 to B05 were used to identify the insect species and samples B01 to B08 were used to identify the plant species (see Results section for details).

**Sampling Interval**	**Collection time per bottle**	**Melbgarten (Bonn)**	**Britz (Eberswalde)**
**1**	31 Mar – 14 Apr 2022	B01	B01
**2**	14 Apr – 28 Apr 2022	B02	B02
**3**	28 Apr – 12 May 2022	B03	B03
**4**	12 May – 26 May 2022	B04	B04
**5**	26 May – 09 June 2022	B05	B05
**6**	09 June – 23 June 2022	B06	B06
**7**	23 June – 07 July 2022	B07	B07
**8**	07 July – 21 July 2022	B08	B08
**9**	21 July – 04 Aug 2022	-	B09
**10**	04 Aug – 18 Aug 2022	-	B10
**11**	18 Aug – 01 Sep 2022	-	B11
**12**	01 Sep – 14 Sep 2022	-	B12
**Total number of samples**		5 insect / 8 plant	12 insect / 12 plant

**Table 2.** Taxonomic information for the insect species shown in Fig. 4. (*) The potential plants pollinated by each species (rightmost column) are matches against the Database of Pollinator Interactions (Balfour et al. 2022) for plants detected at our sampling sites. Note that ‘NA’ indicates where the insect species has been detected, but not any of the corresponding, pollinated plant species.

Image	Order	Family	Species	Author	Sex	Month	Potential plants pollinated according to DoPI (Balfour et al. 2022) (*)
A	Hymenoptera	Vespidae	*Vespulavulgaris*	(Linnaeus, 1758)	female	July	*Boragoofficinalis*, *Callunavulgaris*, *Centaureacyanus*, *Crepiscapillaris*, *Daucuscarota*, *Hederahelix*, *Heracleumsphondylium*, *Pastinacasativa*, *Plantagolanceolata*, *Potentillareptans*, *Trifoliumrepens*, *Tripleurospermuminodorum*
B	Hymenoptera	Apidae	*Bombuspascuorum*	(Scopoli, 1793)	female	August	*Ajugareptans*, *Alliariapetiolata*, *Alliumursinum*, *Bellisperennis*, *Boragoofficinalis*, *Clematisvitalba*, *Crepiscapillaris*, *Daucuscarota*, *Diplotaxistenuifolia*, *Dipsacusfullonum*, *Glechomahederacea*, *Heracleumsphondylium*, *Hypericumperforatum*, *Hypochaerisradicata*, *Lamiumgaleobdolon*, *Leontodonhispidus*, *Lotuspedunculatus*, *Ononisspinosa*, *Papaverrhoeas*, *Prunellavulgaris*, *Ranunculusrepens*, *Stachyssylvatica*, *Torilisjaponica*, *Trifoliumpratense*, *Trifoliumrepens*, *Viciasepium*
C	Coleoptera	Cerambycidae	*Stenurellamelanura*	(Linnaeus, 1758)	male	May	NA
D	Lepidoptera	Pieridae	*Pierisnapi*	(Linnaeus, 1758)	unknown	August	*Bellisperennis*, *Brassicaoleracea*, *Cardaminepratensis*, *Dipsacusfullonum*, *Eupatoriumcannabinum*, *Geraniumrobertianum*, *Glechomahederacea*, *Jasionemontana*, *Prunellavulgaris*, *Ranunculusrepens*, *Syringavulgaris*, *Tripleurospermuminodorum*
E	Lepidoptera	Pieridae	*Pierisrapae*	(Linnaeus, 1758)	unknown	July	*Bellisperennis*, *Brassicanapus*, *Centaureacyanus*, *Crepiscapillaris*, *Eupatoriumcannabinum*, *Prunusavium*, *Tripleurospermuminodorum*
F	Lepidoptera	Nymphalidae	*Parargeaegeria*	(Linnaeus, 1758)	female	July	NA
G	Lepidoptera	Nymphalidae	*Vanessaatalanta*	(Linnaeus, 1758)	female	July	NA
H	Lepidoptera	Lycaenidae	*Polyommatusicarus*	(Rottemburg, 1775)	male	July	*Bellisperennis*, *Callunavulgaris*, *Eupatoriumcannabinum*, *Hypochaerisradicata*
I	Lepidoptera	Noctuidae	*Noctuapronuba*	(Linnaeus, 1758)	unknown	July	*Hederahelix*
J	Diptera	Bibionidae	*Bibiomarci*	(Linnaeus, 1758)	female	April	*Brassicanapus*
K	Diptera	Syrphidae	*Myathropaflorea*	(Linnaeus, 1758)	male	May	*Callunavulgaris*, *Eupatoriumcannabinum*, *Hederahelix*
L	Mecoptera	Panorpidae	*Panorpagermanica*	Linnaeus, 1758	female	April	*Heracleumsphondylium*
